# A Numerical Model for Experimental Designs of Vibration-Based Leak Detection and Monitoring of Water Pipes Using Piezoelectric Patches

**DOI:** 10.3390/s20236708

**Published:** 2020-11-24

**Authors:** Favour Okosun, Mert Celikin, Vikram Pakrashi

**Affiliations:** 1 Dynamical Systems and Risk Laboratory, School of Mechanical and Materials Engineering, University College Dublin, D04 Dublin, Ireland; favour.okosun@ucdconnect.ie; 2 Science Foundation Ireland (SFI) MaREI Centre, University College Dublin, D04 Dublin, Ireland; 3 The Energy Institute, University College Dublin, D04 Dublin, Ireland; 4 Materials Design and Processing Laboratory, School of Mechanical and Materials Engineering, University College Dublin, D04 Dublin, Ireland; mert.celikin@ucd.ie; 5 I- Form, the SFI Research Centre for Advanced Manufacturing, D04 Dublin, Ireland

**Keywords:** PVDF patches, structural health monitoring, sensing, energy harvesting, pipe leak detection, computational fluid dynamics, optimum sensor distribution

## Abstract

While the potential use of energy harvesters as structural health monitors show promise, numerical models related to the design, deployment and performance of such monitors often present significant challenges. One such challenge lies in the problem of leak detection in fluid-carrying pipes. Recent advances in experimental studies on energy harvesters for such monitoring has been promising but there is a paucity in existing literature in linking relevant fluid–structure interaction models around such applications. This paper addresses the abovementioned issue by developing a numerical model with Computational Fluid Dynamics (CFD) and Finite Element (FE) tools and carries out extensive analyses to compare it with existing experiments under controlled laboratory conditions. Conventional Polyvinylidene Fluoride (PVDF) films for leak detection and monitoring of water pipes were considered in this regard. The work provides guidelines on parameter selection and modeling for experimental design and repeatability of results for these types of experiments in future, around the demands of leak monitoring. The usefulness of such models is also demonstrated through the ability to estimate the optimum distribution frequency of these sensors that will enable the detection of the smallest leak of consequence under a known or established flow condition.

## 1. Introduction

Vibration leak detection methods have been identified as effective for early leak detection in pipes. They are a popular choice for any leak detection set-up because they are non-invasive and more suited for monitoring than inspection [1,2,3]. The principle of vibration-based leak detection is anchored to the Fluid–Structure Interaction (FSI) and Negative Pressure Wave Propagation Attenuation Mechanisms (NPWPAM) phenomena [4,5,6]. Research has been carried out using commercially available accelerometers as sensors for vibration pipe leak detection [7,8]. However, there are some established downsides to their use for such applications, ranging from them being costly [9,10], requiring an external power source to operate, to being generally rigid, making it difficult to achieve excellent conformance with the cylindrical pipes they are bonded to [11]. The need for cheap, output-only and flexible vibration sensors for pipe leak detection is what motivated the development of patches made from piezoelectric materials as alternative sensors to commercial accelerometers. PVDF patches are relatively cheaper (when compared to commercial accelerometers), flexible and responsive to leak-induced changes to pipe surface vibration levels. Okosun et al. 2018 [12], presented the fabrication experimental validation for metal pipe leak detection and monitoring of these PVDF patches. Despite the opportunities presented by various experiments, there is a gap in the literature around numerical modeling for such systems. Development of a reasonable fluid–structure interaction model connected to the energy harvesting based monitoring framework can guide future experiments and also help in sensor placement strategies. This paper addresses this gap and puts current experimental results and future experimental designs into context.

Obtaining the pipe surface vibration levels numerically for the healthy pipe state and a number of leak states are essential for experimental design and numerical validation of the PVDF patches for water pipe leak detection, and this task is a two-pronged complex problem. The first phase of the problem deals with the turbulent flow dynamics, Fluid–Structure Interaction (FSI), and leak induced Negative Pressure Wave (NPW; for leak pipe states) with the output from this step being the internal pipe wall pressure fluctuations. The second addresses the propagation of resulting internal pipe wall pressure fluctuation to the external pipe surface exciting vibration response with the output being either pipe external surface strain or acceleration (in this case strain, since piezoelectric patches are strain based vibration sensors). After obtaining the pipe surface strain level for the all the simulation cases, and knowing the properties of the PVDF patches, the theoretical voltage output from the PVDF patches can be calculated for positions of interest along the pipe length, using the already established strain–voltage relationship for PVDF films [13].

In addition to the numerical validation of these piezoelectric patch sensors, this paper also presents a numerical methodology for determining the optimum frequency distribution of these sensors (i.e., their maximum distance apart) that will enable the detection of the smallest conceivable leak of consequence under any known/established flow condition. To be able to detect a small pipe leak under low flow rate conditions using vibration patch sensors, the distance between two sensors must be equal to or less than the length of the portion of the pipe that will be influenced by the induced NPW due to the onset of the leak at that flow condition. Hence, the lowest flow rate, the smallest expected leak size, and the length of pipe influenced by the leak-induced NPW before its complete decay are crucial information for determining the distribution frequency of sensors adopted for a pipe leak vibration monitoring application. Such guidance cannot be obtained from the experimental validation exercise presented in [12] because of the limited size of a typical fluid test rig. The test pipe section of the rig consisted of 100 cm long steel test pipes and the influence of the smallest test leak size (a 2 mm hole) travelled through the entire length of the pipe irrespective of the position the leak was introduced along the pipe length (details of this test rig can be found in [12]).

The results and findings from this paper guide the procedure of creating a numerical framework for interpretation of existing or earlier experiments within the context of the Fluid–Structure Interaction for pipeline leaks in various sectors of application. The work also helps in designing future experiments and provides some quantitative estimates on the choice of parameters for modeling, measuring and comparing along with their quantitative. Finally, the work can be used to obtain the minimum number of sensors required to detect a certain level of leakage, for a given flow-rate. The findings can be easily adapted to a range of sensors and can thus be useful for development of novel sensors and measurement chains around this topic.

## 2. Modeling Turbulent Fluid Flow

### 2.1. Detection Context

Fluid flow regimes are mainly laminar or turbulent [14]. Laminar flow is characterized by fluid particles flowing in orderly streamlines, with each layer moving smoothly past the adjacent layer with little or no mixing, whereas turbulent flow is chaotic where the fluid particles have random motions in all three dimensions. Turbulence leads to irregular and unsteady flow dynamics characterized by the fluctuations of transported quantities (mass, momentum and scalar species), in time and space. In turbulent flow, eddies or vortices are generated by the relative motion of fluids near the boundary layer. These eddies are characterized by identifiable swirling patterns and the energy dissipated by these eddies is converted to heat and wall pressure [15].

The ratio of inertial forces to viscous forces within a flowing fluid, can tell if the flow is laminar or turbulent. This ratio is given by a dimensionless quantity called the Reynolds number (Re) [16], and the relationship is given below.

(1) $$Re=\frac{\rho Ud}{\mu }$$

where ρ is the fluid density, U is the mean velocity of the flow, d is the diameter of the pipe inner cross-section and μ is the fluid dynamic viscosity.

Laminar flows can be described completely mathematically by the continuity, Navier–Stokes, energy conservation equations and the equation of state. However, in the case of turbulent flows, in addition to the aforementioned equations, the turbulence transport properties must also be accounted for [17]. Commercial Computational Fluid Dynamics (CFD) codes provide models that utilizes additional terms (other than those provided by the governing CFD equations) to account for these transport properties but great care has to be taken in the modeling of the problem, which can be a rigorous undertaking. Flows in pipes in real life applications are normally turbulent, which is why they are very complicated [18,19,20]. Fluid Dynamics problems involving flow-induced pressure fluctuations or wall pressure fluctuations caused by turbulence are very complex, often proving difficult to model and solve. It exists over a range of frequencies; hence, it can be termed a broadband phenomenon [21]. This wall pressure fluctuation is the desired output from the first phase of the simulation process in the validation of PVDF patch sensors for pipe leak monitoring and the subsequent section of this paper will provide the details of the solution methodology adopted.

### 2.2. Overview of Modeling Methods

In practice, there are three main methods for the analysis of turbulent flows in commercial CFD codes, namely: Direct Numerical Simulation (DNS), Large Eddy Simulation (LES) and Reynolds-Averaged Navier-Stokes (RANS) models. With DNS being the most accurate and RANS the least accurate.

Direct numerical simulation (DNS) solves these equations numerically in a rigorous way to a desired accuracy without any additional model or correlation. However, its application is still limited because existent problems require a large amount of computational resources, exceeding the capacity of conventional computers, hence it is not a very practical model. For this reason, DNS was not employed in solving the FSI problem of this research [16,18].

Reynolds Averaged Navier–Stokes (RANS) employs equations in modeling the turbulent flow. These models do not provide instantaneous values for the flow and are based on time averages, e.g., they do not compute the pressure fluctuations at the Fluid-Structure Interface [22,23]. The *k–ε* model and the *k–ω* model are the commonly used RANS-based two equations turbulence models. The two extra transport equations accounts for the turbulent properties of the flow. Depending on the chosen model, the transported variables are most often the turbulent kinetic energy k and turbulent dissipation rate ε or specific turbulence dissipation rate ω. The scale and energy of turbulence are determined by solving the two transport equations [24]. The *k–ω* model has an advantage of having an improved performance for near wall boundary layer regions of the flow under adverse pressure gradients when compared to the *k–ε* model. The *k–ε* model on the other hand, is more robust in the free shear flows and mainstream regions [25]. An integrated model that takes advantages of both models is known as the shear stress transport *k–ω* (SST *k–ω*) model [20]. The SST *k–ω* turbulence model operates by employing the *k–ω* model in the near-wall region and by employing a blending function, switches to the *k–ε* model in the free shear flow turbulent region [26].

In this study, internal pipe wall pressure fluctuations or variations is the output quantity of interest from the CFD simulations and it is required as input for the second phase of FE simulations to obtain the pipe surface vibrations, hence, RANS based models cannot accomplish the central purpose of this study. However, the SST *k–ω* RANS model was employed in the process of selection of a mesh for the pipe models before simulation, as one of the selection criteria requires time-averaged solutions.

As described above, the reasons that DNS and the RANS models cannot be employed for obtaining the internal pipe wall pressure fluctuations from the turbulent fluid flow simulation are clear. Here, the LES model, which models the actual physics of the flow better when compared to the RANS models was employed. The LES approach is a hybrid model derived from a combination of DNS and the RANS models. In contrast to a time-averaged approach, LES provides a model that computes the instantaneous velocity and pressure field, in contrast to the time-averaged approach adopted by RANS and it is not as computationally expensive as DNS [16]. In LES, the flow is resolved to a characteristic scale, usually taken to be the size of the grid, and then modeled on the smaller scales. The idea for the LES model stems from the fact that large eddies possess an anisotropic behavior and at the smallest scales, the turbulence is isotropic. Hence, while the large eddies need to be resolved the smallest scales can be solved adequately statistically. Grid scales (GSs) are length scales the size of the grid or larger and scales smaller than that are referred to as subgrid scales (SGSs). The model of a turbulent flow problem should be such that the grid spacing results in most of the total turbulent kinetic energy contained in the large eddies being directly computed, and the remaining fraction of the kinetic energy that is not resolved to the GS modeled [27]. A variety of SGS eddy viscosity models for LES have been detailed in literature including the Wall Adapting Local Eddy-Viscosity (WALE) model, the Smagorinsky and Smagorinsky–Lilly models [16,20]. The LES WALE model was employed for the LES simulation runs in this study since it is known that it performs significantly better in the near wall and boundary layer region when compared to Smagorinsky models [28,29].

In this study, the commercial codes employed were ANSYS FLUENT for the CFD (first phase) simulations and ANSYS Transient Structural for the (FE second phase) simulations. The governing equations and basic formulations of the LES model, which is the primary turbulence model employed in this study can be found in [28,30]. That of the SST *k–ω* RANS model employed in the mesh selection process can be found in [20,26].

### 2.3. Important Parameters in Turbulent Flow Modeling

To get accurate results from the Fluent turbulent simulation, the pipe flow model and simulation set up must at least come close to satisfying certain conditions. There are some parameters that can guide the preparation and validation of the model before it is employed for simulation runs.

#### 2.3.1. Length of the Pipe Domain and Near-Wall Treatment

One of such parameters is the length of the streamwise pipe domain. For a good FLUENT solution, it is advised that the flow should be fully developed. For this to occur, the length of pipe should be at least 5 times the internal or hydraulic pipe diameter [31]. Other important considerations are the inner wall coordinate ($y^{+}$) of the first mesh cell from the pipe wall and fineness of the mesh in the boundary layer regions (near the wall) [32].

(2) $$y^{+}=\frac{u^{*}y}{\upsilon }$$

where $u^{*}$ is the friction velocity at the wall, y is the normal distance from the wall and υ is the kinematic viscosity.

(3) $$u^{*}=\sqrt{\frac{\tau_{w}}{\rho }}$$

where, $\tau_{w}$ is the wall shear stress and ρ is the fluid density.

(4) $$\tau_{w}=0.5C_{f}\rho U^{2}$$

where $C_{f}$ is the skin friction coefficient and U is the average fluid velocity. For pipe flow, $C_{f}$ is given as:

(5) $$C_{f}=0.027Re^{-\frac{1}{7}}$$

To deal with near wall turbulence, the way the near wall flow is treated is important, and this is done using wall functions.

The dimensionless velocity $u^{+}$ is related to the inner wall coordinate, $y^{+}$.

(6) $$u^{+}=F\left(y^{+}\right)$$

(7) $$u^{+}=\frac{u}{u^{*}}$$

where, u is the local velocity.

The laminar sublayer is characterized by small values of $y^{+}$, i.e., $y^{+}<5,$ and in this region the velocity reduces to:

(8) $$u^{+}=y^{+}$$

For larger $y^{+}>30$, the velocity is given as:

(9) $$u^{+}=\frac{1}{\kappa }\ln\left(y^{+}\right)+B$$

where κ=0.419 (Von Karman constant) and B=5.1.

The local $y^{+}$ values determines the layer the first local mesh cell is located and its distance away from the pipe wall, hence the pipe wall treatment applied. For $y^{+}<5,$ the first cell away from the pipe wall is in the laminar sublayer known as the linear region, for $5<y^{+}<30$, it is in the buffer region and for $y^{+}>30,$ it is in the mainstream layer of flow that is predominantly turbulent. This region ($y^{+}>30)$ is known as the log-law layer due to the logarithmic relationship between $u^{+}$ and $y^{+}$. The buffer region is influenced both by the linear and logarithmic regions.

There are two common choices for the wall function: standard wall function and the enhanced wall function. In the standard wall function, the first grid is located within the range $30<y^{+}<150$ and it is in the predominantly turbulent layer. This wall function is employed in simulations where the flow model is large with a very high Reynolds number, making it difficult for the turbulent boundary layer to be resolved due to lack of computer resources. For the enhanced wall function, at least 10 cells should be in the viscosity affected region (laminar sublayer) to be able to resolve it and the first cell should be in the order of $y^{+}\;\mathrm{almost}\;\mathrm{equal}\;\mathrm{to}\;1$ [33].

#### 2.3.2. Mesh Grid Size

To resolve the high energy containing eddies using the LES turbulent model, the mesh of the fluid flow model must be sufficiently fine. Therefore, the meshing of the turbulent flow problem in LES is crucial as it has significant influence on the results. The size of the largest eddies is described by the turbulent length scale, $L_{t}$, in pipe flow problems, hence, $L_{t}$, must be considered in deciding the grid size. $L_{t}$ is approximately 7% of the diameter of the pipe inner cross-section. Eddies of roughly half the size of the turbulent length scale must be resolved to resolve 80% of the turbulent kinetic energy [26]. Hence, the turbulent length scale should serve as a guide in determining the mesh grid size.

#### 2.3.3. LES Time-Step and Courant Number

In selecting transient simulation conditions like the time-step, it is important to consider the characteristic time of transit of a fluid element across a volume. To thoroughly resolve a turbulent flow, the ratio of the time step to the time of transit of the fluid element known as the courant number should be less than 1. The courant number, which provides insights of the fluid movement through the computational cells, is a dimensionless quantity, and can be calculated from:

(10) $$Courant=\frac{\Delta t}{\Delta x_{cell}}U$$

where, Δt is the time step size, U is the average fluid velocity and $\Delta x_{cell}$ is the length of the mesh cell.

A courant number ≤1 means that within one-time step (at most), the fluid particles move from one cell to another. This is the ideal case scenario; hence the time step should be chosen such that courant<1. The two options for reducing the time step if it is greater than one is reducing the time-step and/or coarsening the mesh, if possible.

## 3. Methods

This section first presents an overview of the scope of simulations in the numerical model, identifying the range of flow rates, defining the healthy benchmark and outlining the types of analyses that are carried out subsequently. The validation of the selected mesh is presented next and the wall function of the models is established. Matching of pressure drop through modeling is carried out next, before the final choice of mesh. Considerations of turbulence and mass flow rates due to the leak are considered next. Finally, the vibrations from fluid flow are estimated from the Fluid-Structure Interaction model and subsequently converted to pipe strains, and linked to energy harvesting.

### 3.1. Overview of Numerical Modeling

For the numerical validation of the PVDF patches, the pipe model and simulation conditions were designed to replicate the experimental conditions detailed in [12]. Hence, pipe flow simulations were carried out on 5 states of the pipe i.e., the healthy pipe, 2 mm, 5 mm, 7 mm leak and 10 mm leak states. These simulations were done at 5 different flow rates (ranging from high to low) of 90.85 L/min (24 gpm), 71.92 L/min (19 gpm), 56.78 L/min (15 gpm), 45.42 L/min (12 gpm) and 26.50 L/min (7 gpm), for each pipe state. This resulted in a total of 25 scenarios and independent simulations. Similar to the experimental campaign, the material and properties specified for the pipe model was that of galvanized steel with a length of 1 m. Before the simulation runs, three different pipe mesh models were prepared for the healthy pipe state and a model selection exercise was performed to determine the model, if any, that is best suited for the exercise.

Three healthy pipe models with different mesh models were prepared and an analysis was carried out to validate these meshes and determine the best one to be adopted for the simulation runs. Simulation runs were then performed on the chosen pipe flow model using the LES WALE model of ANSYS FLUENT for the healthy state and leak pipe states of the pipe at various flow conditions. On obtaining the solution of the flow field, the pressure field on the pipe wall was exported to a Finite Element (FE) package (ANSYS Transient Structural) to calculate the pipe response in the form of the pipe surface strain. This pipe surface strain at the positions along the pipe length where the PVDF patch sensors were bonded in the experimental campaign is then used to calculate the theoretical voltage output of the PVDF patch sensors.

### 3.2. Mesh Validation and Selection

Three different pipe mesh models were prepared for the healthy pipe state and a model selection exercise was performed to determine the model, if any, that is best suited for the exercise. The validation exercise was carried out for the healthy pipe state only, at all 5 test flow rates, therefore, it involved 5 simulation scenarios out of the 25 total scenarios. In addition to the important modeling parameters mentioned in the previous section, a comparison of the static pressure gradients (ΔP) from each validation simulation and all three models with the theoretical pressure gradient obtained using the Darcy-Weisbach equation was employed in the mesh selection process. The Darcy-Weisbach equation, given below, relates the pressure loss, due to friction along the length of the pipe to the average velocity of the fluid flow for an incompressible fluid.

(11) $$\frac{\Delta P}{L}=f\frac{\rho U^{2}}{2D}$$

where L is the length of the pipe, D is the internal pipe diameter, U is the mean flow velocity and f is the Darcy friction factor, obtained from the Colebrook equation given below:

(12) $$\frac{1}{\sqrt{f}}=-0.869\ln\left(\frac{e_{roughness}}{3.7D}+\frac{2.523}{Re\sqrt{f}}\right)$$

where $e_{roughness}$ is the pipe wall roughness, which is equal to $1.5\times 10^{-4}\;m$ for the galvanized steel pipe material.

The closer the FLUENT pressure gradient is to the theoretical pressure gradient for the different test scenarios, the more suitable the mesh model is. The Darcy–Weisbach equation is only valid for the steady state fully developed pipe flow, hence the SST *k–ω* RANS model was employed for the mesh selection exercise as LES models are employed for transient simulations. This section of the paper discusses the pipe model and mesh preparation, the conditions of the simulations, the setup for the SST *k–ω* RANS model employed for this exercise, and comparison of the simulation results of the three mesh models.

The operational pipe model and meshing was done in ANSYS ICEM CFD. ICEM CFD provides advanced geometry/mesh generation and mesh diagnosis and repair functions necessary for the in-depth analysis [34]. The pipe material was galvanized steel with properties presented in Table 1 below. Bearing the important turbulent model parameters in mind, three mesh models of the healthy pipe were prepared such that each model had cell elements starting at different distances from the pipe wall. The models also had varying sizes of the mesh grid and different mesh growth rate as you move from the pipe wall to the main turbulent flow stream. Simulations were performed on these models using the SST *k–ω* RANS model at the 5 different flow rates.

For the setup of each model, the fluid domain was set to be water (density of $1000\;\mathrm{kg}/m^{3}$ and dynamic viscosity of $1.0\times 10^{-3}\;\mathrm{Ns}/m^{2})$. The velocity-inlet Boundary Condition (BC) was selected for the pipe inlet, the value of velocity of flow is calculated from the flow rate for each of the simulation scenario. The pressure outflow BC was selected for the pipe outlet. This boundary condition was selected over the pressure outlet BC because it allows Fluent to calculate the pressure gradient (ΔP) along the pipe length without imposing a pressure value at the outlet, which the pressure outlet BC requires. This provided a better representation of the experimental setup that we are hoping our model replicates. This is because, in the experimental validation setup [12], the test pipe outlet does not discharge to the atmosphere, as the water returns back to the reservoir and is circulated in a cyclic manner, hence, we cannot specify a gauge pressure of zero as the outlet pressure. Additionally, although there is a pressure gauge at the outlet of the test pipe section of the test rig, the resolution of the gauge and errors associated with such measuring devices means that whatever pressure read-out by the gauge is only an approximation of the actual outlet pressure. So, since the output of interest was not in the point-by-point static pressure along the pipe length but rather the pressure gradient along the length of the pipe, it was decided that it was best to use the pressure outflow BC and allow Fluent calculate this pressure gradient. Stationary wall with “no slip” was employed for the pipe wall.

Table 2 below provides details of the mesh of the three candidate mesh models being evaluated. From the table, HEX cells and QUAD faces refers to meshing elements hexahedral cells and quadrilateral faces, respectively. The table shows that mesh model 3 had the finest meshing with the most nodes and boundary faces while mesh model 1 had the coarsest meshing.

**Figure 1:**
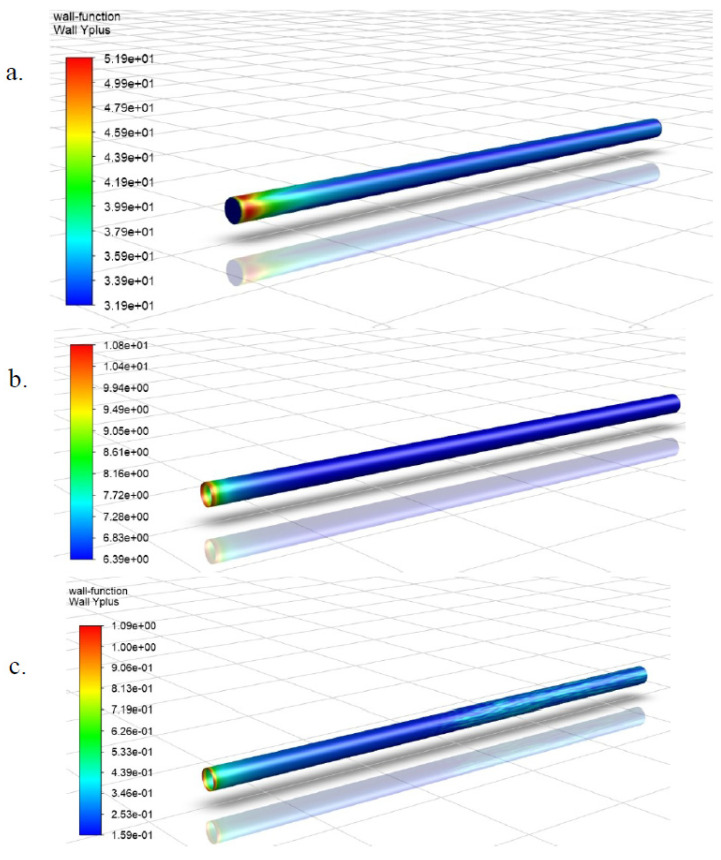
Wall $y^{+}$ contours at highest test flow rate of 90.85 L/min for: (**a**) Mesh Model 1; (**b**) Mesh Model 2 and (**c**) Mesh Model 3.

After running the simulations for the 3 models at all 5 flow rates, the wall function of the models was established from the simulation results and this was the first consideration in the mesh selection process. The wall $y^{+}$ values are directly proportional to the average velocity of flow (see Equation (2)), hence results from the simulation at the highest flow rate of 90.85 L/min will be employed for ascertaining the maximum possible $y^{+}$ values and the wall-function applied by the candidate models. The contours of wall $y^{+}$ for the pipe models is shown in Figure 1 below.

From Figure 1 above, Mesh 1 had $y^{+}$ values in the range of about 31–51, meaning that the closest cells to the pipe wall for this mesh is in the turbulence dominant region of the flow, hence, near wall treatment was what was applied for this model. Mesh 2 had $y^{+}$ values in the range of about 6.5–11, meaning that the closest cells to the pipe wall were in the buffer region, and Mesh 3 had values in the range of about 0.16–1, meaning that the closest cells to the pipe wall was in the laminar sublayer, and consequently satisfying the conditions for enhanced wall treatment, even at the highest simulation flow rate. Based on these findings, Mesh 3 best satisfied this selection criteria as the objective was to implement an enhanced wall treatment wall function in the model to better resolve the turbulence close to the pipe wall and obtain more accurate wall pressure fluctuations.

**Figure 2:**
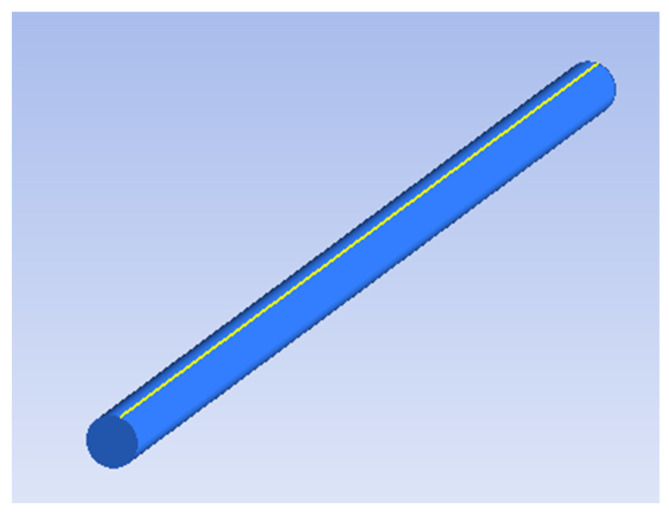
Straight line through the centre of the internal pipe wall.

**Figure 3:**
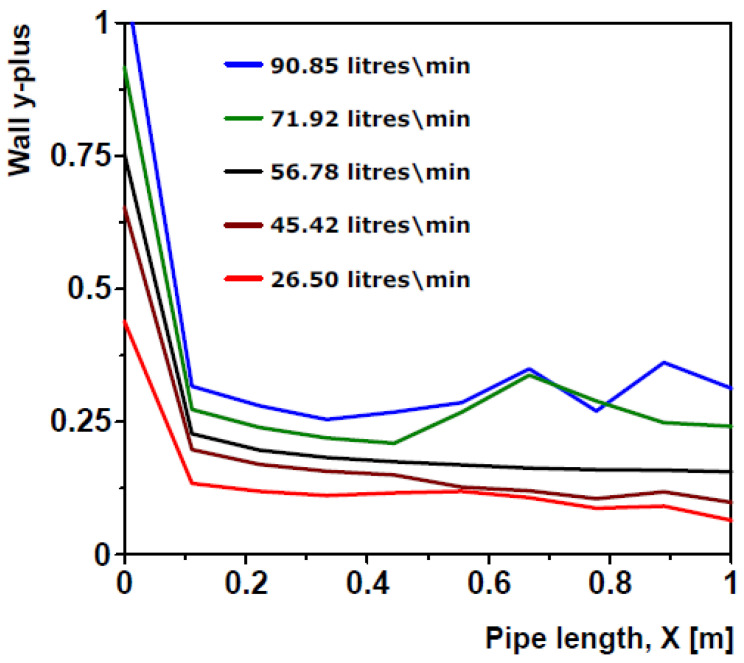
Wall $y^{+}$ curves for mesh model 3 at all simulation flow rates.

A straight line through the centre of the internal pipe wall along the entire pipe length is shown in Figure 2. Figure 3 subsequently presents curves of wall $y^{+}$ for model 3 and all 5 test flow rates. These curves show the wall $y^{+}$ values recorded along below.

Figure 3 shows that the range of $y^{+}$ values recorded along the pipe wall for model 3 decreased with decreasing mean flow velocity, justifying the earlier assertion that the maximum possible $y^{+}$ values will be obtained from the simulation at the highest flow rate. Additionally, it can also be seen from the figure that the $y^{+}$ values was highest at the pipe inlet and it fell steeply just after the pipe inlet as you move along the pipe length until it became almost stable. This is because $y^{+}$ values tend to be highest in the developing regions of the flow, hence it is highest at the uniform velocity pipe inlet and decreases as the velocity profile develops [35].

### 3.3. Consideration of Pressure Drop

**Figure 4:**
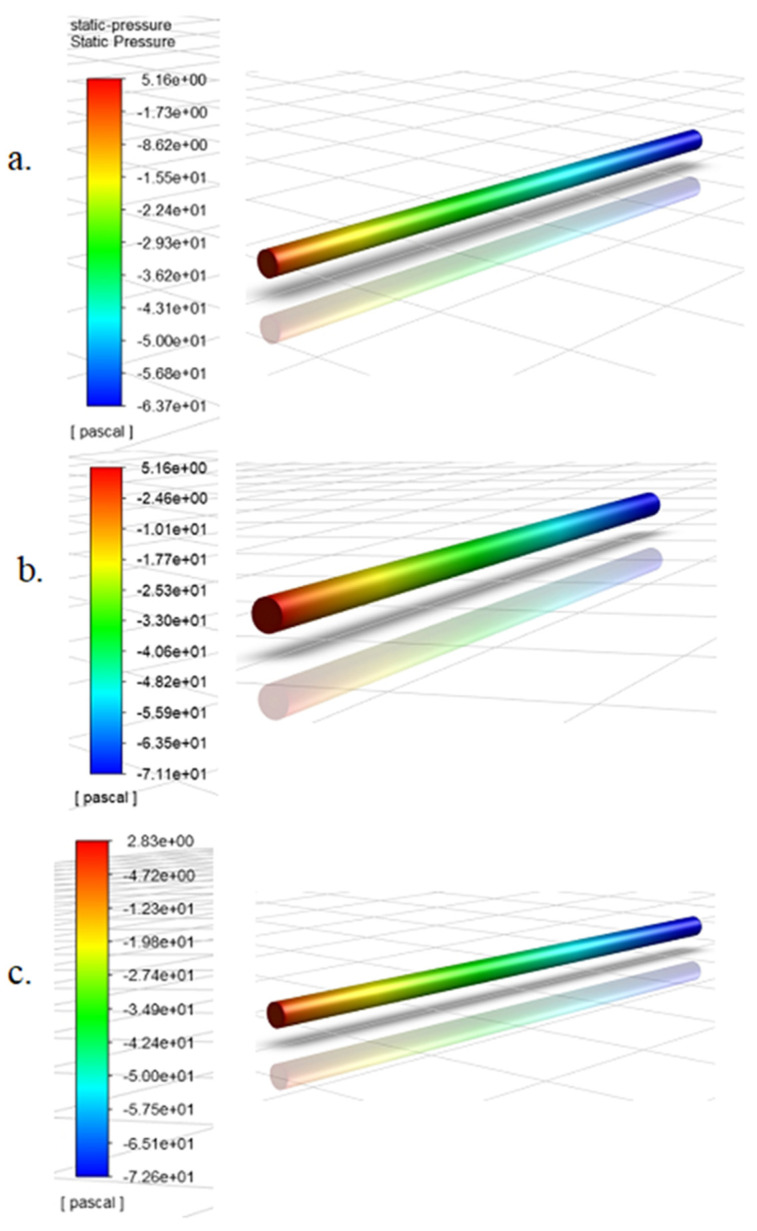
Static pressure contours at the lowest simulation flow rate of 26.50 L/min for: (**a**) Mesh Model 1; (**b**) Mesh Model 2 and (**c**) Mesh Model 3.

The second consideration in this selection exercise involved calculating the theoretical pressure drop (ΔP) due to friction along the pipe length for the 5 flow rates from Equations (11) and (12), and comparing the values to the pressure gradient obtained for the three models for the 5 simulation scenarios. In the calculation of the theoretical pressure gradient, the Darcy friction factor, f was first solved for implicitly using Equation (12). Figure 4 below shows representative contour diagrams from the lowest simulation flow rate for all three candidate models.

From these contours, the fluent pressure drop was calculated for all test flow rates (including the higher flow rate simulation cases whose contour diagrams were not presented). The table below shows a summary of the theoretical and Fluent pressure gradients for all simulation cases and candidate models.

Table 3 presents the theoretical pressure gradients (Theo. ΔP) for all the flow rates, the simulation pressure gradients (FLUENT ΔP) for the three candidate mesh models, andquantifies the percentage deviation (in parenthesis) of the FLUENT ΔP from the theoretical ΔP for all simulation cases and mesh models. From the table, the simulation pressure gradients obtained from the results of mesh model 3 had the least deviation from the theoretical pressure gradient for all simulation cases while Mesh 1 consistently registered the highest deviation. The values from mesh 3 closely matched the theoretical values that the maximum deviation recorded was under 1%. The deviation for mesh 2 was consistently higher than that of mesh 3, but less than that of mesh 1. Another noteworthy feature of the table is that the Darcy friction factor (f) slightly increased with decreasing flow velocity. This is because f is inversely proportional to the Reynolds number. However, this does not translate to an increase in the pressure drop (ΔP) with decreasing flow rates or Reynolds number. The reality is quite the contrary, as (ΔP) will continue to decrease with decreasing flow rates, because the flow velocity and turbulence that is directly proportional to ΔP has a higher influence on frictional losses than the friction factor, f. ΔP varies directly with the second power of the fluid velocity and the first power of f (see Equation (11)). Additionally, the friction factor is analogous kinetic or sliding friction, which is much less than static friction [36,37]. Table 3 also shows the Reynolds number and friction factor values at each simulation flow rate, the Reynolds numbers were calculated from Equation (1) and it shows that the flow is turbulent (Re >4200) for all simulation cases.

### 3.4. Choice of Mesh

**Figure 5:**
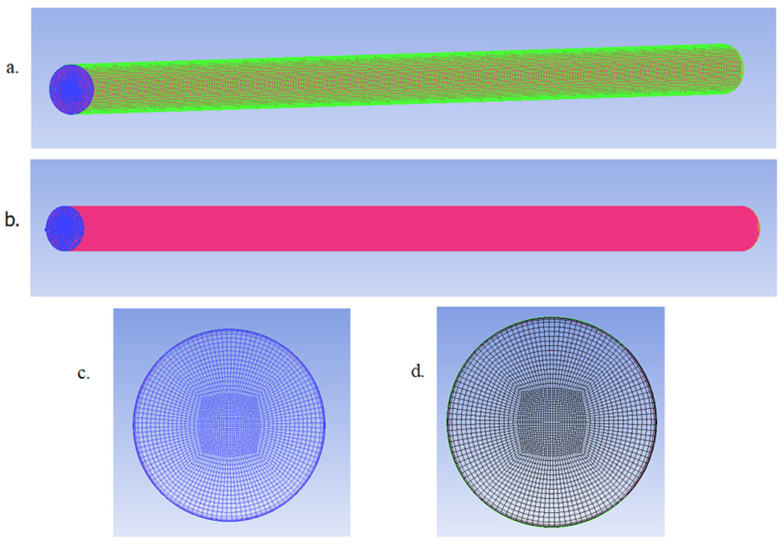
Selected pipe mesh model (mesh 3) showing: (**a**) the internal pipe wall and inlet; (**b**) fluid domain (pipe wall suppressed); (**c**) pipe inlet and (**d**) pipe outlet.

From the result of the mesh validation exercise, it is clear that mesh 3 (the finest mesh) satisfied both selection criteria best and will afford the best opportunity of obtaining accurate results from the simulations. The performance of mesh 2 was fair, but it did not satisfy the enhanced wall treatment condition and the difference in the number of cells and nodes when compared to mesh 3 was not large enough to present a substantial gain in computation time. Hence, mesh 3 was selected for this exercise. Figure 5 below presents simple wire-frame display of the selected mesh model.

A close look at Figure 5c,d shows a concentration of mesh cells close to the pipe wall. It is also worthy to note that the largest cell in the selected mesh model, i.e., model 3, had a maximum volume of $1.165\times 10^{-9}\;m^{3}$, which gives a maximum length dimension of $1.05\times 10^{-3}\;m$. The recommendation for the maximum length of a node as stated earlier is that it should not be more than half of the turbulent length scale, $L_{t}$ = 0.07D. For this pipe flow model, half of $L_{t}$ was $1.31\times 10^{-3}\;m$, which was more than the maximum length of any cell in the mesh model. This further validated the selected model as ideal for this exercise.

### 3.5. LES Setup to Obtain Internal Pipe Wall Pressure Fluctuations

After selection of the mesh model, the first phase for the validation of the PVDF sensors was executed using the LES WALE turbulence model. As earlier stated, the LES turbulent model is the best practical model for solving transient turbulence problems. This phase involved running simulations for the healthy state pipe and damaged state pipes for all 5 flow rates to obtain the internal wall pressure fluctuations due to FSI for the healthy state pipe and FSI and NPW for the leak state pipe. The idea here is that the influence of the leak will cause an increase in the pipe pressure fluctuation due to the leak induced NPW, and this increase in NPW will in turn reflect as an increase in the pipe surface strain for the leak state pipe when compared to the healthy state pipe at the same flow rate.

For the simulations, the healthy pipe model was that of the selected mesh model and the BCs also remained the same as those employed in the mesh selection exercise. For the leak states of the pipe, leak being represented as 2 mm, 5 mm, 7 mm and 10 mm diameter circular holes were introduced at the 60 cm mark along the pipe length of the selected model without really changing the other model mesh properties, the only modification from the healthy state pipe model is that the mesh nodes closer to the leak was made finer. The leaks have a small leak wall of 2.5 mm, which is the same as the thickness of the test pipe, this is because they were modeled such that they represent the physical pipe leak state as best as possible. Consequently, in addition to the inlet, outlet and pipe wall boundaries, there were two additional boundaries, namely the leak wall, and the leak outlet. The BC for the leak wall was the same as the pipe wall, i.e., stationary wall with no slip, and mass flow outlet BC was employed for the leak outlet. Since the leak discharges to the atmosphere, atmospheric condition was selected as the operating condition at the leak outlet. The velocity-inlet and pressure outflow BCs remained the pipe inlet and pipe outlet BCs.

The theoretical leak mass flow rates $\left(Q_{l}\right)$ employed for the simulations were derived from the leak orifice equation presented in Equation (13) below [38].

(13) $$Q_{l}=\frac{C_{K}A_{K}\sqrt{\rho \left[8A^{2}({\bar{P}}_{0}-\left(\frac{l}{L_{p}}\right)\left({\bar{P}}_{0}-\;{\bar{P}}_{L_{P}}\right)+C_{K}{}^{2}A_{K}{}^{2}\rho a^{2}\right]}-C_{K}{}^{2}A_{K}{}^{2}\rho a}{2A}-2\frac{A}{a}P_{g}$$

where $C_{K}$ and $A_{K}$ are the discharge coefficient and area of the leakage orifice respectively, *A* is the area of the pipe inner cross-section, ρ is the density of fluid in the pipe and a is the speed of propagation of the NPW in the pipe medium. ${\bar{P}}_{0}$ and ${\bar{P}}_{L_{P}}$ are the steady pressures at inlet and outlet of the pipeline before leakage respectively, $L_{P}$ is the length of the pipeline, l denotes the distance of the leakage site from the inlet and $P_{g}$ denotes the pressure relative to barometric pressure around the outside pipe wall (which is zero in this case as the leak discharges to the atmosphere).

The relationship for calculating a in the pipe medium can be found in [38], and was determined as 1.383 km/s. For pipe flow, $C_{K}=0.6$ [39]. Table 4 below shows the leak mass flow rate values for all the simulation pipe flow rates and pipe leak states.

**Figure 6:**
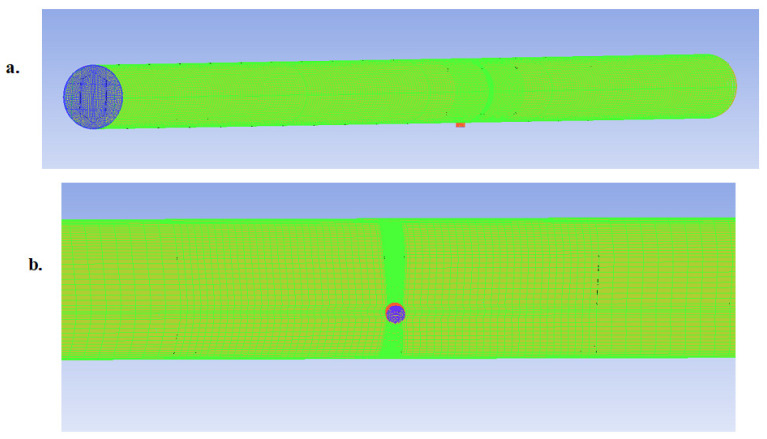
A 5 mm leak pipe mesh model showing: (**a**) the full pipe length and (**b**) the leak area.

Figure 6 below shows a representative mesh for the leak pipe states with a focus on the leak area.

For the LES simulations, a time-step size of 0.001 s was selected, and the simulation was run for 2000 time-steps for each simulation case. This amounted to a total flow time of 2 s. The courant number criterion was used to determine if the selected time-step size and mesh model was adequate for this transient simulation exercise. As mentioned earlier, for a good solution the Courant number is less than one. Using the above transient conditions, the healthy pipe simulations were first conducted for all 5 flow rates to determine the courant number.

Similar to the wall $y^{+}$, the courant number is also directly proportional to the average velocity of flow (see Equation (10)), hence results from the simulation at the highest flow rate of 90.85 L/min will give the highest courant number range and thus will be employed in evaluating the chosen time-step size and pipe mesh model.

**Figure 7:**
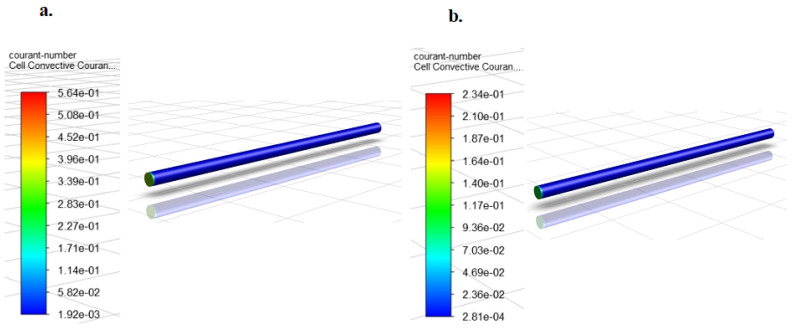
Courant number contours for healthy pipe transient simulations at (**a**) the highest simulation. Flow rate of 90.85 L/min and (**b**) the lowest simulation flow rate of 26.50 L/min.

Figure 7 above shows that the range of values of courant number from the simulation is between 0.00192 and 0.564 at 90.85 L/min and 0.000281 and 0.234 L/min at 26.50 L/min. These representative flow rates were the highest and lowest simulation flow rates. From these results, we could tell that the maximum obtainable courant number from all the simulation cases when employing the selected pipe mesh model and time-step size was 0.564, which was less than 1. This validated the chosen time-step size of 0.001 s and the selected pipe mesh model.

Additionally, another noteworthy point is that the range of values (maximum and minimum) obtained from the courant number contours for the simulation at the highest flow rate was higher than that recorded for the lowest flow rate, confirming that courant number was directly proportional to the average flow velocity. This justifies the decision to use the highest flow rate courant number contour for validating the time-step and pipe mesh model.

After the successful validation of the transient simulation conditions, the simulations were conducted for all the pipe states (healthy and leak states) at all 5 flow rates using a time-step size of 0.001 s, and 2000 time-steps. This amounted to a total of 25 simulation cases. The average time to complete one simulation ranged from 26 to 30 h. During the simulation, the data sampling for time statistics was turned on, and set to every time step, meaning that all 2000 time-steps were employed in calculating the transient results from the FLUENT simulation, After the simulations have been completed, the fluctuating pressure of the internal pipe wall was then extracted and exported into a transient finite element model of the pipe to obtain the external pipe surface strain conditions for each pipe state and flow condition.

### 3.6. Determination of Pipe Surface Strain Conditions and Theoretical PVDF Patch Voltage Output

Although all 2000 time-steps were employed in calculating the FLUENT transient results, the pressure fluctuations of the internal pipe wall were extracted for every 10 time-steps for each simulation case and imported into the pipe FE model in ANSYS Transient Structural to determine the structural response (pipe surface strain fluctuation) of the pipe using ANSYS CFD-Post. This meant that a total of 200 time-step pressure fluctuation results were imported into ANSYS Transient Structural per simulation case. This is because extracting these results is very laborious and time consuming as it must be done one time-step at a time for each case. There is also the problem of file size restrictions when saving extracted data. The extraction of the file was done with the aid of the record session option in ANSYS CFD-post.

Before importing the pressure fluctuations, the FE pipe model was set up and validated. For the model set up, after meshing, the pipe support type was specified to be fixed supports as both inlet and outlet boundary conditions. The distance between these supports being the length of the test pipe, i.e., 1 m. This best represents the physical condition of the test pipe during the experimental validation exercise (as it was clamped to the test rig at both inlet and outlet) [12]. The pipe material was specified to be galvanized steel.

After validating the FE model, it was adopted for the transient structural simulations. As earlier stated, the pressure fluctuations were imported into the transient structural model for all 25 simulation cases from FLUENT. The pipe surface strain response was recorded and the theoretical performance of the PVDF sensor was evaluated for positions of interest along the pipe length, employing the PVDF film sensor–voltage relationship established by [13] and shown below. Table 5 below presents a summary of the fabricated sensor properties. The relationship between the strain acting on the piezoelectric patch and the resulting voltage is presented as

(14) $$\varepsilon_{1}=\frac{V_{p}C_{P}}{S_{q}}$$

where $\varepsilon_{1}$ is the strain acting on the sensor, $V_{p}$ represents the voltage generated by the sensor, $C_{P}$ is capacitance of the sensor and $S_{q}$ is a sensitivity parameter = $d_{ij}YA_{p}$. Additionally, *d_ij_* is the piezoelectric constant, *Y* = Young’s modulus of the Piezoelectric material and $A_{p}$ = Area of the sensor.

### 3.7. Determination of the Optimal Distribution of PVDF Patches to Detect the Smallest Pipe Leak

Ensuring that there is optimum distribution of vibration sensors along the pipe length for any pipe leak vibration monitoring application is very important. If the distance between the sensors is much less than what is optimal to detect the smallest expected leak size, although leak detection might be achieved, it will lead to deploying more sensors than what is required thereby driving costs up. This will have a significant financial impact in extensive applications involving many sensors. On the other hand, the distance between the sensors being more than what is optimal will affect the performance of the monitoring system negatively, as small leaks might not be detected. This paper attempts to establish a simple method for determining this optimum sensor distribution. Here, we relied on CFD modeling using ANSYS FLUENT to achieve this by modeling the smallest leak in the pipe model adopted for the numerical validation of the PVDF patches and running simulations for the lowest operating flow rate. Since leak detection is reliant on transient leak induced NPW altering the pipe surface strain conditions, the length of the area of pipe affected by this NPW obtained from simulations based on the above leak size and flow rate conditions, can be adopted as the maximum allowable distance between two sensors.

To demonstrate this idea, we took the pipe model and simulation conditions employed in the preceding subsection of this paper for validating PVDF patch sensors for leak detection into consideration. The lowest flow rate in this case was 26.50 L/min and the smallest leak size 2 mm. The pipe model being a 1 m long galvanized steel pipe. From the results obtained, it was clear that that the influence of the small leak at that flow rate was prominent throughout the entire pipe length, because although the leak-induced NPW started decaying away from the leak, it still had positive values both at the pipe inlet and pipe outlet (more details on this is provided in the next section), this shows that the pipe length was too short to determine the maximum distance between two sensors to detect the smallest leak size of 2 mm at the lowest pipe flow rate.

**Figure 8:**
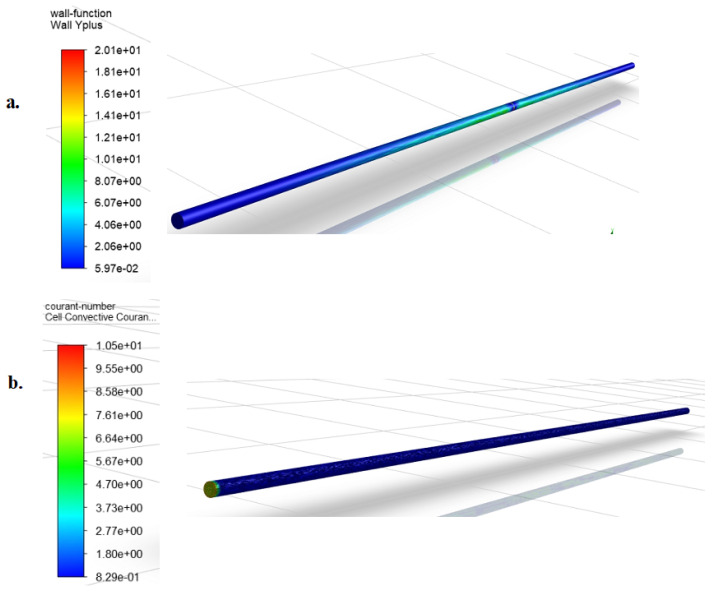
Contours of turbulent parameters for a 4 m length pipe model showing: (**a**) wall $y^{+}$ and (**b**) courant number.

Here, the solution to this problem was attempted by creating a healthy pipe mesh model with the same internal diameter but a longer length. The mesh of this model was validated following the steps employed in the Section 3.1. By observing the trend of decay of the leak induced NPW at the simulation case of interest (i.e., 26.50 L/min flow rate and 2 mm leak size) from the validation exercise results, a pipe length of 4 m was deemed sufficient for this investigation. The BCs and model setup adopted was the same as those employed in Section 3.1. A bid to satisfy the conditions of $y^{+}\approx 1$ and courant<1 that was met in the preceding subsection resulted in too many mesh nodes due to the 4 m length of the pipe, and an attempt at simulation kept crashing FLUENT. Care was taken to mesh the model with the above turbulent parameter conditions relaxed a bit. This resulted in an effective mesh with wall $y^{+}$ range of 0.0597–10.5, indicating the first cells from the pipe wall were partly in the buffer layer and the laminar layer. The courant number was found to range from 0.829 to 10.50. These can be seen from the Figure 8 below.

Similarly, the 2 mm leak pipe model was also created, and it had the same properties as the healthy pipe model, except for the additional leak outlet and leak wall BCs. This time, the leak was introduced at the half-way along the pipe length, i.e., 2 m from the inlet and outlet. The BCs employed here was also the same as that employed in the preceding Section 3.2. FLUENT simulations were run for both the healthy and leak pipe models, and the results were analyzed to determine the length of pipe influenced by the leak induced NPW before it completely decayed, and consequently, the optimum distance between two PVDF patch sensors for the subject pipe model and flow conditions.

## 4. Results and Discussion

This section demonstrated the impact of the model developed in this paper. The simulations first established the root-mean-square estimates of negative pressure waves in pipes to be a valid indicator of the leak. The effect of presence, location and the extent of leakage and its interaction with the distance from the sensor were investigated next extensively for various flow rates and levels of leakage. The effective calibrations of markers of such detection are presented, which is particularly relevant for any future experimental design. The section subsequently demonstrated how the spacing of the sensors could be determined through the developed numerical method as a function of the smallest size of leak that is intended to be detection.

### 4.1. Numerical Validation of PVDF Sensor Patches

#### 4.1.1. Pipe Flow Simulations (FLUENT)

Post Fluent simulation, the pipe wall fluctuating pressure for the simulation time, at any point along the pipe length of the pipe, could be obtained. The frequency of sampling being the inverse of the time-step size, hence 1000 Hz. The instantaneous pipe wall fluctuating pressure ($P_{f}$), at any point along the pipe length and at any time of sampling, t is given by the relationship below (Bai et al., 2019).

(15) $$P_{f}=P-P_{m}$$

where P is the instantaneous static pressure and $P_{m}$ is the mean static pressure calculated over the sampling time.

**Figure 9:**
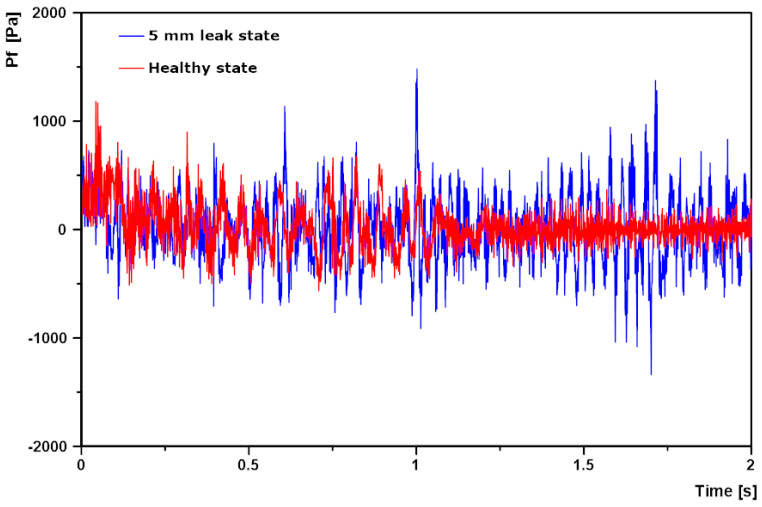
Plots of $P_{f}$ against time, for the healthy pipe state and 5 mm leak pipe state at 90.85 L/min.

When $P_{f}$ plotted against time, the result is a random curve with alternating negative and positive values. Plots of $P_{f}$ against time for a point at the 0.6 cm mark along the pipe length just above the leak for a two of the simulation cases is presented in Figure 9 below. The figure shows that for the same flow condition and position on the pipe, the 5 mm leak pipe state recorded higher amplitude of $P_{f}$ over the simulation flow time than the healthy pipe. This is because the $P_{f}$ is solely a consequence of FSI alone for the healthy pipe, while it is due to FSI plus NPW for the leak pipe states.

From the FLUENT simulation results, time-averaged statistics was calculated for all simulation cases. One of those statistics important to this analysis is the Root Mean Square Error (RMSE) of the static pressure. The RMSE of static pressure is the same as the root mean square of the fluctuating pressure $\left(P_{f}rms\right)$. The calculated rms fluctuating pressure can be obtained for any point along the pipe length, and it provides a single representative value for the magnitude of the time varying pressure fluctuation recorded at that point over the entire simulation time. That way $P_{f}$rms along any line running through the pipe wall can be plotted against the pipe length. The value of $P_{f}$rms can be obtained for as many points as possible. This is not possible for experimental data, which can only be recorded in positions where the sensors are bonded. Here, $P_{f}$rms is recorded along a straight line through the centre of the internal pipe wall along the entire pipe length (see Figure 2), for all simulation cases. For each flow condition, the difference in the $P_{f}$rms obtained for each pipe state and that obtained for the healthy pipe state at that same flow rate was calculated. This represents the contribution of the *NPW* to the fluctuating pressure recorded for that simulation case. Here we refer to this difference as the NPWrms.

**Figure 10:**
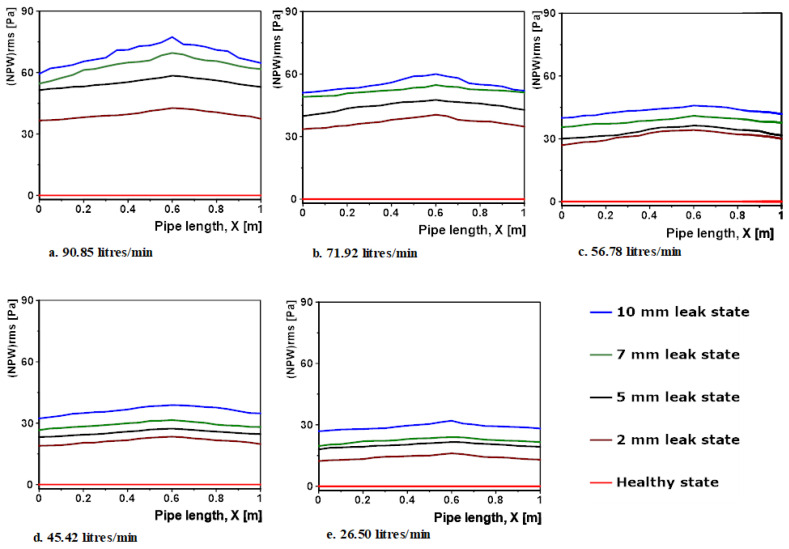
Plots of  NPWrms recorded along the pipe length for all simulation cases for flow rates (**a**) 90.85 litres/per minute (**b**) 71.92.85 litres/per minute; (**c**) 56.78 litres/per minute; (**d**) 45.42 litres/per minute; (**e**) 26.50 litres/per minute.

Figure 10 below shows plots of NPWrms against the pipe length (X). In the figure, each subfigure shows curves of NPWrms for the different pipe states at a common flow rate. From the plots, it can be seen that in all cases of the healthy pipe, there was no NPW, this is expected as the healthy pipe states are the baseline conditions. For all the leak pipe states, the influence of the  NPW is prominent throughout the pipe length, even at the furthest sections from the leak (inlet and outlet), the *NPW* have positive values for all simulation cases. This is the case for the smallest leak size 2 mm at the lowest flow rate that unsurprisingly records the least NPWrms curve in terms of magnitude. An important trend to notice is that the NPW was consistently highest at the leak position (i.e., 60 cm mark), and in all simulation cases it gradually decayed in both directions away from the leak in an almost identical manner. Furthermore, observing individual subfigures, it was observed that the value of *NPW* with decreasing flow rates, i.e., the curves for 90.85 L/min had the highest magnitudes and 26.50 L/min the least magnitudes. This is because as the flow rate increased, the flow turbulence increased and consequently the FSI. Additionally, flow also had a directly proportional relationship with the leak induced *NPW*.

The findings from this analysis served to confirm that an NPW was induced on the onset of a leak, this NPW increased with increasing leak size and it contributed to the internal pipe wall pressure fluctuation of any leaking pipe. Therefore, monitoring any parameter that was influenced by this *NPW* could prove an effective method for leak detection and monitoring.

#### 4.1.2. Transient Structural Simulations and Determination of Theoretical PVDF Patch Output

**Figure 11:**
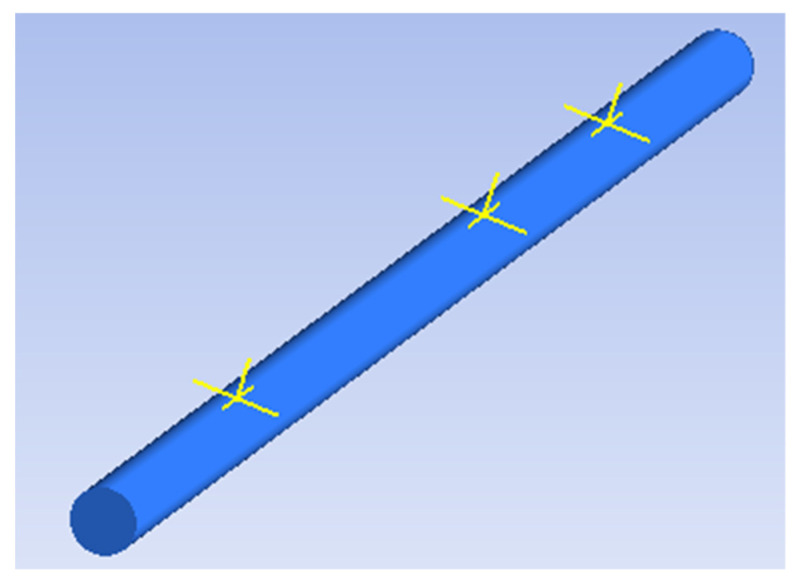
The three PVDF patch sensor positions from the pipe inlet.

Post transient structural simulations, the pipe vibration response in terms of surface strain had to be obtained at specific positions along the pipeline to be able to calculate the theoretical voltage output from PVDF patch sensors. The positions selected were 20 cm, 60 cm and 80 cm from the pipe inlet, named sensor position 1 (SP1), sensor position 2 (SP2) and sensor position 3 (SP3) for the purpose of analysis (see Figure 11). Sensor position 2 (SP2) was directly above the leak.

**Figure 12:**
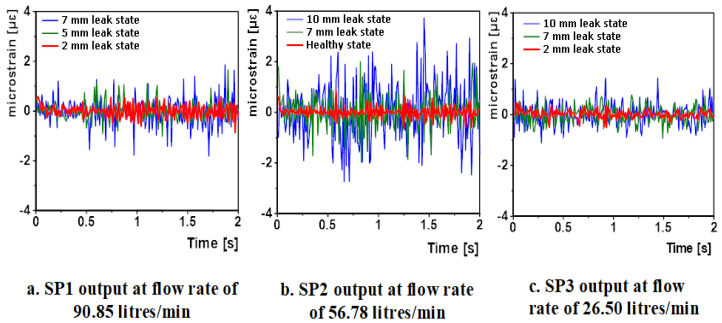
Samples of strain data extracted at SP1, SP2 and SP3 for a number of simulation cases, at different flow rates (**a**) 90.85 litres/per minute; (**b**) 56.78 litres/per minute; (**c**) 26.50 litres/per minute.

These are the same positions where the patches were bonded for the experimental campaign detailed in [12]. The pipe surface strain conditions at these three points were extracted for all simulation cases and the data adopted for analysis. Figure 12 below shows representatives of this data.

The above figure shows that at a given flow rate, the amplitude of the recorded strain increased with the introduction of a 2 mm small leak and this amplitude increased even further with increasing leak severity.

**Figure 13:**
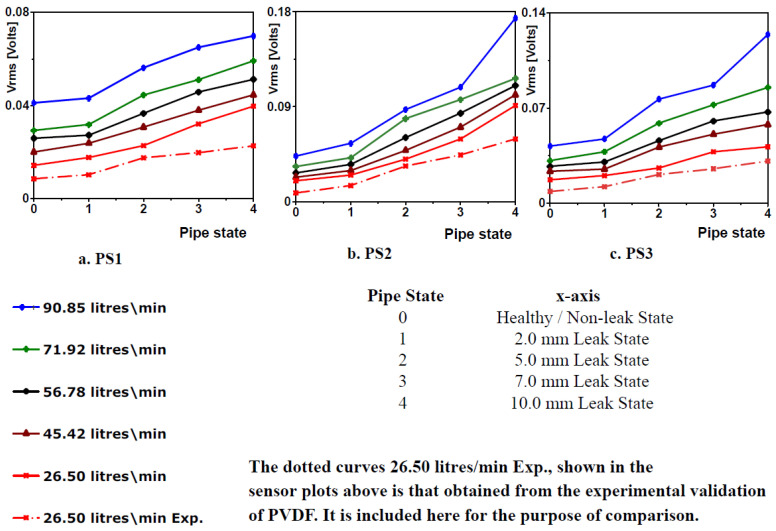
Theoretical Vrms output of PVDF patches test scenarios (**a**) PS1; (**b**) PS2; (**c**) PS3 with the locations of sensors indicated as SP1, SP2 and SP3 respectively in Figure 12.

To determine the theoretical performance of the PVDF patch sensors, the theoretical root mean square voltage, Vrms from the sensor for each of the three sensor positions and all simulation cases must be calculated. This is the same analysis adopted for the experimental validation. Additionally, it was the only way to see if the trend of the theoretical and experimental sensor outputs was in tandem. To do this, the root mean square of the strain data was first calculated for all simulation cases and sensor positions. Then using the established voltage–strain relationship for PVDF patches (Equation (14)), the theoretical Vrms for each sensor was calculated and it is presented in the Figure 13 below. The sensors are named PS1, PS2 and PS3 in line with sensor positions 1, 2 and 3. They were also named this way for the experimental validation exercise [12].

The horizontal (x) axis from Figure 13 below represented the health of the pipe, where 0 is the healthy pipe state, 1 is the 2 mm leak pipe state, 2 is the 5 mm leak pipe state, 3 is the 7 mm leak pipe state and 4 is the 10 mm leak pipe state.

It is important to note that, the Vrms values of the pipe states simulated for are represented on the plots by markers (see figure legend). The Vrms values of the intermediate pipe states inferred by joining the markers to form curves do not represent data from this exercise as simulations were not conducted at these intermediate states. Joining the markers to form curves is for visual aid, to help appreciate the trend of the theoretical sensors’ output with the worsening pipe leak state.

From Figure 13, the introduction of 2 mm leak resulted in an increase in the voltage output (Vrms) of the PVDF sensors. This can be observed by the upward tilt of the curves moving from points 0–1 on the horizontal axis representing the state of the pipe, and with increasing severity of the leak, there is a corresponding increase in the sensor voltage output. This is depicted by the rise in the curves moving from left to right of the plots. This shows that at a given flow rate, the pipe experiences more vibration with the introduction of leaks due to the induced NPW and furthermore vibration as the leak size increased. This increasing vibration was reflected consistently from the simulation results and consequently in the theoretical output of all the PVDF patches.

The trend of the curves obtained from the results of this numerical validation exercise explained above mirrors that obtained from the experimental validation exercise detailed in [12], and both numerical and experimental results validates PVDF patches as effective for pipe leak detection and monitoring. The disparity being that the theoretical Vrms of the PVDF sensors was consistently higher than the experimental output of the sensors. This is the case for all simulation cases and sensor positions. Included in Figure 13 plots are the curves from the experimental results of the 26.50 L/min test scenarios, for illustration and comparison. The intention was to show more curves from the replica experimental results in the above figure, but due to almost coincident points and curves crossing, the idea was shelved. The representative experimental 26.50 L/min curves were lower in magnitude when compared to their theoretical counterparts for all three sensors, and it is a representative of the relationship between the theoretical and experimental curves of the other flow rates (see [12] for plots of curves from the experimental sensor output). The maximum deviation was observed for PS1 at a simulation test flow rate of 26.50 L/min and 10 mm leak pipe state, where the theoretical Vrms was 1.92 times the experimental Vrms. There might be a few reasons for this, one being that there are likely small measuring losses through the piezoelectric measuring chain, which may lower the experimental Vrms. Another one is that although, there are no vibration losses in the finite bond layer thickness between PVDF patch sensors and the pipe if perfectly bonded [13], any imperfection, however small, while adhering the patches to the pipe surface will result in little losses, and this only affects the experimental results as no correction factor was applied to the strain–voltage relationship when calculating the theoretical Vrms. A final important consideration is that, although a lot of consideration and checks were implemented in executing this numerical validation exercise to ensure reliable simulation results, the fluid flow turbulence problem is such a complex one that a numerical solution that approximates and gives verifiable information about the practical flow solution can be deemed reliable.

Additionally, since the leak induced NPW that aids leak detection was highest at the leak position and decayed away from the leak in both directions, the area of the pipe closest to the leak could be identified, by monitoring the leak induced vibration at various sections of the pipe. The fact that the sensors were at different distances from the leak position was utilized in evaluating the theoretical performance of the PVDF sensors for leak localization. The approach employed here is same as that employed in the experimental validation [12], where the leak induced vibration is quantified by a theoretical leak index that measures how much the theoretical Vrms for a PVDF sensor patch at a particular flow rate deviates from the healthy state theoretical Vrms at the same flow rate.

**Figure 14:**
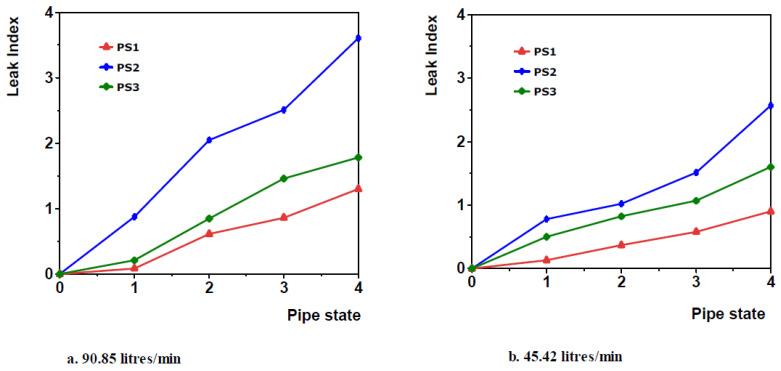
Representative lots of the theoretical leak index against the pipe state at two flow rates (**a**) 90.85 litres/per minute and (**b**) 45.42 litres/per minute.

The leak index was calculated for all simulation cases and the three PVDF patch sensors. Figure 14 below show the plots of theoretical leak index against pipe state for the three sensors at two different flow rates.

The horizontal (x)-axis representation is same as that employed for Figure 14. The plots in Figure 14 show that the theoretical leak index curves for PS2, which was bonded just above the leak was the highest while that of PS1, which was 40 cm from the leak was the lowest at both flow rates. The curves for PS3 located 20 cm from the leak were in-between those of PS2 and PS1 at both flow rates shown. This is the same for all flow rates, and consistent with the trend of the experimental leak index presented in [12].

The results numerically validate the experimental results from the PVDF patch sensors obtained in [12], as the theoretical results and performance indicate that they could detect, monitor and localize a leak.

### 4.2. Determination of the Optimal Distribution of PVDF Patches to Detect the Smallest Pipe Leak

As mentioned earlier, the healthy pipe and 2 mm leak pipe states at a flow rate of 26.50 L/min simulation conditions from the numerical validation exercise were adopted for this study. The 2 mm leak pipe state at flow rate of 26.50 L/min simulation condition corresponds to the smallest leak pipe state at the lowest flow rate from the numerical exercise. For this study, the length of the pipe model was 4 m as opposed to the 1 m length from the numerical validation study. Figure 10e shows the NPWrms curve for 2 mm leak size at 26.50 L/min for the numerical validation study. From the curve, we can see that the influence of the small leak at that flow rate was prominent throughout the entire 1 m pipe length, because although it started decaying away from the leak, it still had positive values both at the pipe inlet and pipe outlet, this shows that the 1 m pipe length was too short to determine the maximum distance between two sensors to detect the smallest leak size of 2 mm at the lowest pipe flow rate. This justifies using a longer pipe for this study, and by observing the trend of decay of the NPW in Figure 10e, a pipe length of 4 m was deemed sufficient for this investigation.

**Figure 15:**
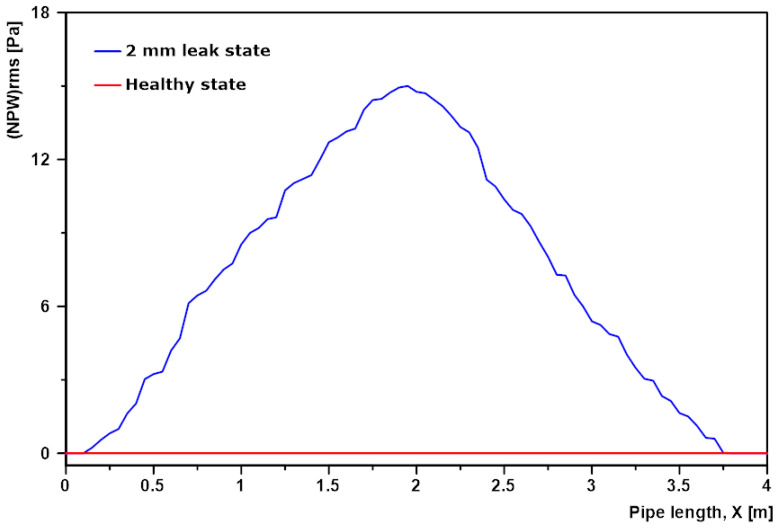
Plot of NPWrms for the 2 mm leak pipe state at 26.50 L/min.

After FLUENT simulations, the $P_{f}$rms curves along a straight line (similar to Figure 2), on the internal pipe wall was obtained for healthy pipe state and the 2 mm leak pipe state. The NPWrms curve of the 2 mm leak state pipe was derived by subtracting the $P_{f}$rms curve of the healthy pipe from the $P_{f}$rms curve of the leak pipe state. Plot of NPWrms against pipe length is shown in Figure 15 below.

From Figure 15 above, the value of NPW was zero along the pipe length for the healthy pipe state, because the healthy pipe is the baseline condition and there was no leak inducing NPW. For the 2 mm leak pipe state the NPW was at its highest at the position of the leak, i.e., the 2-m mark along the pipe length. This NPW gradually decayed in both directions away from the leak finally falling to zero at the 0.15 m mark and the 3.75 m mark. The area under the curve represents the area of the pipe affected by the NPW induced due to the 2 mm smallest leak size and the least simulation flow rate of 26.50 L/min. The length of pipe in this area was 3.60 m and it represents the maximum distance between two piezoelectric strain-based sensors if the 2 mm leak was to be detected at a flow rate of 26.50 L.

Therefore, for the pipe model in this exercise and our simulation condition, the maximum distance between two sensors for the smallest leak size to be detected at the least operating pressure was approximately 3.60 m.

The presented numerical framework and related experiments could thus adapt itself to not just detection hardware [40] but also for other output-only detection or monitoring frameworks [41,42,43], degradation [44] or understanding of computing demands [45].

## 5. Conclusions

This paper presented a numerical model, combining the Fluid–Structure Interaction to estimate the vibrations and dynamic strains on a fluid-carrying pipe and estimated the impact of leaks. The work then linked this model to energy harvesting based monitoring of such leaks through a PVDF patch, in the context of existing experimental results. This method relies on monitoring the leak induced NPW and its attenuation away from the leak. The results from the numerical validation exercise corroborated the experimental results presented in [12], providing a first comprehensive bench marked evidence base and implementation protocol on this topic. The model was then able to estimate the maximum spacing of sensors that still can detect the minimum leak of consequence. The results provided guidelines for future experimental designs through the type model presented here and established sensor placement strategies. The model can be adapted easily to new sensors and detection algorithms and can thus be used for assessing the performance of a sensor or a method in future. The results from the exercise reinforced the confidence in piezoelectric patch sensors as being effective for pipe leak detection and monitoring.

Despite these advantages, the study naturally has some limitations. The energy-harvesting model is relatively simplistic and variations of harvesting circuits have not been explored, limiting the discussions to the open circuit voltages. The electromechanical coupling is kept constant, but in low powers, this coefficient may vary. The challenge of potential false alarms caused by environmental perturbations unrelated to the leak has not been investigated here either. Additionally, effects of temperature, chemical exposure, humidity and material degradation and durability have not been considered in this model.

## Figures and Tables

**Table 1:** Properties of the pipe material.

Parameter	Value	Unit
Internal pipe diameter	37.3	mm
Pipe wall thickness	2.5	mm
Bulk Modulus	160	GPa
Modulus of Elasticity	200	GPa
Poisson ratio	0.29	NA
Density	7850	kg/m^3^

**Table 2:** Mesh description of candidate models.

Mesh	Fluid Domain	Interior Faces	Inlet Faces	Outlet Faces	Wall Faces
	(HEX Cells)		(QUAD Faces)		
Model 1	496,545	1,187,992	1754	1754	21,368
Model 2	671,553	1,897,520	2697	2697	7652
Model 3	787,089	2,343,664	3161	3161	38,884

**Table 3:** Comparison of theoretical and simulation pressure gradients.

Flow Rate	Re	f	Theo. ΔP	FLUENT ΔP (Pa) Outlet Faces		
(liters/min)			(Pa)	Mesh 1	Mesh 2	Mesh 3
90.85	51,686.80	0.0305	785.05	751.26 (4.30%)	777.62 (0.94%)	783.88 (0.15%)
71.92	40,918.72	0.0309	498.46	462.52 (7.21%)	483.00 (3.10%)	499.61 (0.23%)
56.78	32,304.35	0.0315	316.74	287.88 (9.11%)	303.27 (4.26%)	317.94 (0.38%)
45.42	25,843.47	0.0322	207.23	193.91 (6.43%)	213.80 (3.17%)	209.13 (0.92%)
26.50	15,075.35	0.0342	74.90	68.85 (8.07%)	76.26 (1.81%)	75.43 (0.71%)

**Table 4:** Leak mass flow rate for all leak pipe state simulation cases.

Flow Rate	$\mathrm{Leak}\;\mathrm{Mass}\;\mathrm{Flow}\;\mathrm{Rate},\;Q_{l}\;(\mathrm{Kg}/s)$ Outlet Faces			
(liters/min)	2 mm Leak	5 mm Leak	7 mm Leak	10 mm Leak
90.85	0.0054	0.026	0.053	0.11
71.92	0.0045	0.021	0.042	0.086
56.78	0.0028	0.016	0.033	0.068
45.42	0.0021	0.013	0.027	0.054
26.50	0.0013	0.0079	0.016	0.032

**Table 5:** PVDF patch sensor properties.

Parameter	Value	Unit
Area, A_p_	0.00175	m^2^
Capacitance, C_p_	Approx. 3.30	nF
Thickness, t_p_	52	µm
Resistance, R	2.66	MΩ
Modulus of Elasticity, Y	8.3	GPa
Piezoelectric strain constant, d_31_	30	PC/N
